# A functional IFN-λ4-generating DNA polymorphism could protect older asthmatic women from aeroallergen sensitization and associate with clinical features of asthma

**DOI:** 10.1038/s41598-017-10467-y

**Published:** 2017-09-05

**Authors:** Sreedhar Chinnaswamy, Aleksandra Wardzynska, Malgorzata Pawelczyk, Joanna Makowska, Tea Skaaby, Josep M. Mercader, Tarunveer S. Ahluwalia, Niels Grarup, Marta Guindo-Martinez, Hans Bisgaard, David Torrents, Allan Linneberg, Klaus Bønnelykke, Marek L. Kowalski

**Affiliations:** 1 National Institute of Biomedical Genomics, PO:N.S.S, Kalyani, 741251 West Bengal India; 2 Dept. of Immunology, Rheumatology & Allergy, Medical University of Lodz, Lodz, 92-213 Poland; 3 Department of Rheumatology, Medical University of Lodz, 92-003 Lodz, Poland; 4 Research Centre for Prevention and Health, the Capital Region of Denmark, Copenhagen, Denmark; 5 Barcelona Supercomputing Center (BSC). Joint BSC-CRG-IRB Research Program in Computational Biology, 08034 Barcelona, Spain; 6 COPSAC, Copenhagen Prospective Studies on Asthma in Childhood, Herlev and Gentofte Hospital, University of Copenhagen, Copenhagen, Denmark; 7 Chair of Clinical Immunology and Microbiology, Healthy Aging Research Center, Medical University of Lodz, 251 Pomorska Str, 92-213 Lodz, Poland; 8 Department of Clinical Experimental Research, Rigshospitalet, Denmark; 9 Department of Clinical Medicine, Faculty of Health and Medical Sciences, University of Copenhagen, Copenhagen, Denmark; 10 Institució Catalana de Recerca i Estudis Avançats (ICREA), Barcelona, Spain; 11 The Novo Nordisk Foundation Center for Basic Metabolic Research, Faculty of Health and Medical Sciences, University of Copenhagen, 2100 Copenhagen, Denmark

## Abstract

Lambda interferons (IFNLs) have immunomodulatory functions at epithelial barrier surfaces. IFN-λ4, a recent member of this family is expressed only in a subset of the population due to a frameshift-causing DNA polymorphism rs368234815. We examined the association of this polymorphism with atopy (aeroallergen sensitization) and asthma in a Polish hospital-based case-control cohort comprising of well-characterized adult asthmatics (n = 326) and healthy controls (n = 111). In the combined cohort, we saw no association of the polymorphism with asthma and/or atopy. However, the IFN-λ4-generating ΔG allele protected older asthmatic women (>50 yr of age) from atopic sensitization. Further, ΔG allele significantly associated with features of less-severe asthma including bronchodilator response and corticosteroid usage in older women in this Polish cohort. We tested the association of related *IFNL* locus polymorphisms (rs12979860 and rs8099917) with atopy, allergic rhinitis and presence/absence of asthma in three population-based cohorts from Europe, but saw no significant association of the polymorphisms with any of the phenotypes in older women. The polymorphisms associated marginally with lower occurrence of asthma in men/older men after meta-analysis of data from all cohorts. Functional and well-designed replication studies may reveal the true positive nature of these results.

## Introduction

Type III interferons (IFNs) or IFN-λs (or IFNLs) are known to play critical roles in innate and adaptive immune responses to viral infections. Several recent reports have implicated IFN-λs as the ‘guardians’ of the epithelial barrier surfaces that encompass large regions of the human body that include respiratory, gastrointestinal and reproductive tracts^1^. Single nucleotide polymorphisms (SNPs) at the *IFNL* locus on human chromosome 19 were discovered a few years ago to be associated with chronic hepatitis C virus (HCV) infections^2–4^. These SNPs, discovered initially by genome-wide association studies (GWAS) to be associated with treatment-induced clearance of HCV, got widely validated and were subsequently found to be associated with different human diseases, both viral and non-viral in origin (reviewed in 5). The immunomodulatory role of IFN-λs is thought to be the molecular mechanism behind these associations^5^. Among the different SNPs at the *IFNL* locus, that are in strong linkage disequilibrium (LD) in most populations, a dinucleotide polymorphism rs368234815 (TT/ΔG) has emerged as the ‘causal’ variant^5, 6^. The ΔG allele of the variant causes a shift in the open reading frame of the *IFNL4* gene, giving rise to a new IFN-λ called IFN-λ4^6^. IFN-λ4 has undergone purifying selection during human evolution with highest frequency of the functional IFN-λ4-generating ΔG allele in the African population (0.78) and lowest in the East Asian population (0.06)^6, 7^; 50-60% of the European population carries at least one copy of the functional gene^8^. IFN-λ4 is a potent antiviral cytokine with structural and functional similarity to IFN-λ1, 2 and 3^8, 9^. The latter are known to modulate adaptive immunity favoring a Th1 predominant response^10–12^. Even though there is no direct evidence yet, IFN-λ4 is also expected to participate in shaping and maintaining innate and adaptive immune responses at the epithelial lining of the respiratory tract owing to its similarities with other IFN-λs. The interactions of IFN-λs with the newly discovered innate lymphoid cells (ILCs)^13^ are likely to hold the key to improving our understanding of diseases like allergy and asthma^5^.

Atopy, defined as increased predisposition to generate specific IgE to common allergens, and diagnosed in an individual subject by presence of aeroallergen sensitization, may lead to Th2-driven immune response to harmless allergens resulting in development of allergic diseases including asthma. Exacerbations in asthma are commonly associated with respiratory virus infections, and impaired innate immune responses have been documented in asthma patients^14, 15^. Since both the development of atopic predisposition and asthma exacerbations may involve respiratory viral infections, we undertook this study with the hypothesis that these complex disorders are influenced by the IFN-λ4-generating polymorphism.

## Results and Discussion

### ΔG allele of rs368234815 protects older women asthmatics from atopy in a Polish hospital-based case-control cohort

We used a Polish discovery cohort that included 326 well-characterized adult bronchial asthmatics recruited from the university asthma clinic, medical university of Lodz. Cases were heterogeneous with respect to the disease control and severity. Controls comprised of 111 volunteers without any chronic respiratory disorders representing a sample of general population. Information on atopic sensitization (determined by skin prick test or SPT to a panel of inhalant allergens) was available for 384 subjects (273 asthmatics and 111 controls). The patient and control group characteristics are shown in Table 1. Owing to its functional nature we chose and genotyped the functional IFN-λ4-generating polymorphism rs368234815 in this cohort. Power calculations showed that we had enough sample size to give >80% power (at a significance level of p = 0.05) to detect an effect size of 1.55 and 1.6 in atopy and asthma, respectively.

**Table 1:** Characteristics of the study subjects in the Polish cohort.

	asthma, N = 326	controls, N = 111
age, years*	**59.2 ± 15.9 (18–94)**	**52.3 ± 18.3 (24–81)**
gender, women, n (%)	201 (61.7%)	72 (64.9%)
Men > 50 yr average age (range)/n	67.3 ± 9.8 (50–87)/92	65.2 ± 9.7 (50–80)/18
Men < 50 yr average age (range)/n	34.2 ± 7.5 (18–48)/33	32.9 ± 5.9 (24–48)/21
Women > 50 yr average age (range)/n	67.3 ± 9.3 (50–94)/144	67 ± 7.2 (51–81)/47
Women < 50 yr average age (range)/n*	**40 ± 6.6 (24–49)/57**	**34.1 ± 6.5 (26–49)/25**
Atopy, n/N tested (%)*	**141/273 (51.6%)**	**39/111 (35.1%)**
FEV1% pred.	75.3 ± 24.1 (17.5–129.5)	–
FEV1%/FVC	68 ± 13.1 (25.6–99.4)	–
ACT, points	17.8 ± 5.5(4–25)	–
FeNO (ppb)	30.6 ± 17.8 (2–184)	–
Asthma control (according to GINA 2016)		
Controlled, n (%)	72 (22%)	–
partly controlled, n (%)	96 (30%)	–
Uncontrolled, n (%)	156 (48%)	–
Current asthma treatment		
ICS, n (%)	240 (73.6%)	–
low dose**, n (%)	28 (8.6%)	
medium dose**, n (%)	120 (36.8%)	
high dose dose**, n (%)	92 (28.2%)	
Oral steroids, n (%)	23 (7.1%)	–
LABA, n (%)	197 (60.4%)	
Leukotriene antagonists, n (%)	64 (19.6%)	
at least 1 exacerbation/last year, n (%)	170 (52.1%)	–

Values are presented as arithmetic means + SD, (range); *statistically significant difference between groups, p < 0.05; values shown in bold; **according to GINA 2016.

The Minor Allele Frequency (MAF) in the combined population of controls and asthma patients (cases) was 0.34; the distribution of genotypes was: 38%, 53%, 9% and 44%, 44%, 12% for TT/TT, TT/ ΔG and ΔG/ΔG in controls and cases respectively. Both controls and cases were in Hardy-Weinberg equilibrium (HWE) (P > 0.05) either individually or as a combined group. In the combined group analysis there was no association of the polymorphism with asthma or atopy under either dominant or recessive models of inheritance (Table 2). However, by using log-linear regression we observed statistically significant interactions between the polymorphism, atopy, age and gender (interactions up to three factors; polymorphism, age and gender p = 0.037; polymorphism, gender and atopy p = 0.035). To dissect these interactions further, we stratified our atopy data into several groups based on age and gender (Table 3) and applied bonferroni correction to avoid false positives arising due to multiple testing. Since our study group had a majority of women (Table 1), we chose 50 years as a divider of age in the combined cohort for our stratified analysis as it is also the age of attainment of menopause^16^ (overall median age at natural menopause in Poland is 51.25 years^17^,). Only among the >50 yr sub-group of women (older women) we saw a significant association of the polymorphism with atopy after multiple testing correction under a dominant model of inheritance for the minor allele. The ΔG allele conferred protection from allergic sensitization in older asthmatic women. The significance of association was retained in both the asthmatic older women and in the combined group of asthmatic and control older women, but not in the control older women’s sub-group when tested alone, in both univariate and multivariate analysis (Table 4).

**Table 2:** Association of the functional IFN-λ4-generating polymorphism rs368234815 with asthma and atopy in the Polish cohort.

	Genotype (n, %)				**Dominant model** (TT/ΔG + ΔG/ΔG) vs TT/TT	**Recessive model** ΔG/ΔG vs (TT/ΔG + TT/TT)
	TT/TT	TT/ΔG	ΔG/ΔG	Total	OR (95% CI); p-value	OR (95% CI); p-value
**Asthma**	145, 44.5	142, 43.5	39, 12	326, 100	0.75 (0.48–1.18); 0.26	1.37 (0.66–2.85); 0.48
**Controls**	42, 37.8	59, 53.2	10, 9	111, 100		
**Atopy +**	81, 45	80, 44.4	19, 10.6	180, 100	0.72 (0.48–1.09); 0.14	0.84 (0.44–1.59); 0.63
**Atopy −**	76, 37.2	103, 50.5	25, 12.3	204, 100		
Asthma*						
**Atopy+**	64, 45.4	61, 43.3	16, 11.3	141, 100	0.75 (0.46–1.22); 0.27	0.81 (0.39–1.66); 0.58
**Atopy−**	51, 38.6	63, 47.7	18, 13.7	132, 100		
Controls						
**Atopy+**	17, 43.6	19, 48.7	3, 7.7	39, 100	0.68 (0.31–1.52); 0.41	0.77 (0.18–3.17); 1
**Atopy−**	25, 34.7	40, 55.5	7, 9.7	72, 100		

*Skin prick test was not carried out for 53 asthmatics as they were under treatment with antihistamines, antidepressants or there were other contraindications.

**Table 3:** The ΔG allele of rs368234815 associates with protection from atopy in older women in the Polish cohort.

Group	Sub-group	N	OR (95% CI)	p-value	p-value*
All	All ages	384	0.72 (0.48–1.09)	0.123	1
	>50 yr	261	0.51 (0.31–0.85)	**0.009**	0.081
	<50 yr	123	1.26 (0.59–2.68)	0.548	1
Men	All ages	145	1.28 (0.66–2.47)	0.458	1
	>50 yr	94	0.81 (0.35–1.88)	0.394	1
	<50 yr	51	1.53 (0.41–5.73)	—	—
Women	All ages	239	0.51 (0.3–0.86)	**0.012**	0.108
	>50 yr	167	0.37 (0.19–0.72)	**0.002**	**0.018**
	<50 yr	72	1.01 (0.39–2.62)	1	1

*After bonferroni correction; A dominant model of inheritance for the minor allele (TT/ΔG + ΔG/ΔG vs TT/TT) was used to compute OR.

**Table 4:** Association of the functional IFN-λ4-generating polymorphism rs368234815 with atopy in older women in the Polish cohort.

Group	Dominant Model (TT/ΔG + ΔG/ΔG) vs TT/TT					
	OR (crude)	95% CI	p	OR (adjusted)*	95% CI	p
^1^Women (Asthma) > 50 yr N = 128; atopy, n = 55	**0.398**	**0.188–0.841**	**0.016**	**0.402**	**0.184–0.877**	**0.021**
^2^Women (Controls) > 50 yr N = 39; atopy, n = 11	0.400	0.098–1.631	0.202	0.366	0.083–1.605	0.171
^3^Women (Asthma + Controls) > 50 yr N = 167; atopy, n = 66	**0.379**	**0.198–0.724**	**0.003**	**0.409**	**0.208–0.803**	**0.016**

*^1^, *^2^ for age; *^3^ for asthma status and age. A dominant model of inheritance is shown with significant results (p < 0.05) in bold. No significant association under any group/sub/group was seen using the recessive model of inheritance.

Even though the association was significant in the older women’s sub-group, small sample sizes may have prevented us from appreciating the effect of the polymorphism on atopy in the remaining sub-groups. In addition, a validation of the observed effect on the older women’s sub-group is required from other populations and/or geographical regions. To examine this and to investigate the association of the *IFNL* locus SNPs with atopy, asthma and related illnesses, we used data from the Genetic Epidemiology Research on Adult Health and Aging (GERA, dbGaP Study Accession: phs000674.v1.p1)^18^, Inter99^19^, Health2006^20^ and COPSAC^21, 22^ cohorts. The characteristics of the different study cohorts are briefly described in Table 5. The GERA cohort consisted of predominantly older participants (average age 63 yr, range 18 to over 100 yr) and hence all participants in this cohort were considered as being older (>50 yr) for analysis.

**Table 5:** Characteristics of the different study cohorts used in the study.

Cohort	Type	Genotype* tested	MAF	Phenotype data available	Total no. of participants	Ref.	Remarks
Inter 99	Population-based	rs12979860 (g)	0.34	Atopy Asthma	5341	19	Atopy determined by serum specific IgE; self-reported doctor-diagnosed asthma
Health 2006	Population-based	rs12979860 (g)	0.34	Atopy Asthma	3134	20	
COPSAC	Case-control (hospital-based﻿﻿;﻿ parents of asthmatic children)	rs12979860 (g)	0.30	Atopy Asthma	551	21	All participants below 50 yr of age; Atopy determined by serum specific IgE
GERA	Population-based	rs12979860 (i); rs8099917 (i)	0.32; 0.19	Allergic rhinitis, Asthma	56637	18	Average age 63 yr (range18 to over 100 yr)
Polish	﻿Hospital-based Case-control	rs368234815 (g)	0.34	Atopy, asthma	437	—	Atopy determined by SPT; asthma treated and monitored

*g-genotyped; i-imputed; MAF-minor allele frequency.

**Figure 1:**
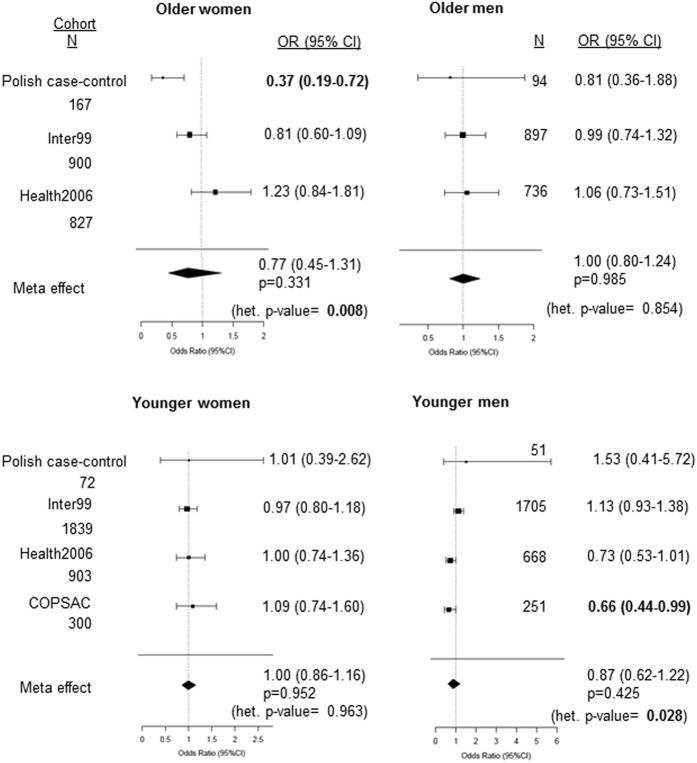
Forest plots showing association of *IFNL* polymorphisms and atopy in sub-groups based on age and gender in different cohorts. The tag-SNP rs12979860 was used in all other cohorts except the Polish cohort that tested for functional polymorphism rs368234815. A dominant model of inheritance for the minor allele (Ex. TT/ ΔG + ΔG/ ΔG vs TT/TT for rs368234815) was used in all cohorts except in the COPSAC cohort that used an additive model to obtain Odds Ratios (OR) shown as a forest plot. p-value < 0.05 was considered as significant and is in bold. For stratification based on age 50 yr was used as a cut-off mark. Het.-heterogeneity.

Genotype information for a related *IFNL* polymorphism rs12979860^5^ was available in the other European cohorts, hence, we tested the association of atopy with rs12979860 in the Inter99, Health2006 and the COPSAC cohorts in four different sub-groups based on both age and gender and did a meta-analysis using a random-effects model on all the cohorts where atopy data was available including the Polish cohort (Fig. 1). Even though, the Polish cohort tested for rs368234815 and the other cohorts for rs12979860 the data could be compared since a strong LD (r^2^ = 0.98) between them in the European population has been recorded^6^; furthermore, the MAFs in the cohorts were similar (Table 5). Moreover, recent data shows that the SNP rs12979860 is the ‘best tag-SNP’ for the functional polymorphism rs368234815 due to a common underlying linkage structure at the *IFNL* locus^23^. The results show that we failed to replicate our findings from the older women’s sub-group of the Polish cohort, in other cohorts (Fig. 1). Further, except for a nominal association (p = 0.04) with an additive genetic model in the COPSAC younger men’s cohort no other sub-group in any of the remaining cohorts showed any significant association with atopy (Fig. 1). The older women’s and younger men’s sub-groups had significant heterogeneity in the effect of the polymorphisms (p = 0.008 and p = 0.028 respectively) between the different cohorts and no significant association was seen after meta-analysis. Even in the other sub-groups where there was no significant heterogeneity between the studies, no significant effect of the polymorphism on atopy was noted in the meta-analysis (Fig. 1). Similar results were seen using the fixed-effect model (Suppl. Figure 1)

**Figure 2:**
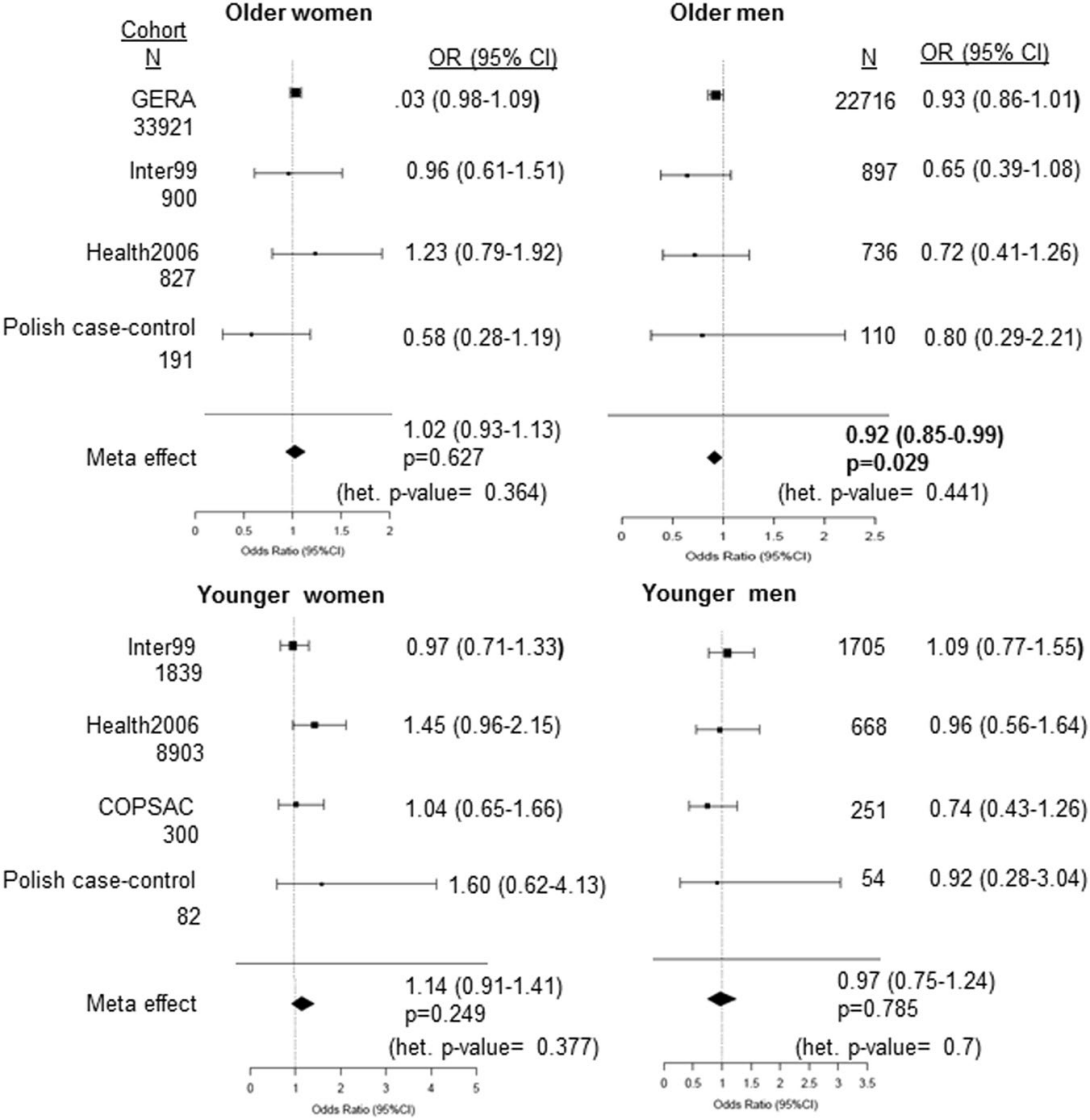
Forest plots showing association of *IFNL* polymorphisms and asthma in sub-groups based on age and gender in different cohorts. The polymorphisms tested are similar to Fig. 1. A dominant model of inheritance for the minor allele (Ex. TT/ ΔG + ΔG/ ΔG vs TT/TT for rs368234815) was used in all cohorts to obtain Odds Ratios (OR) shown as a forest plot. p-value < 0.05 was considered as significant and is in bold. Meta-analysis was performed using a random-effects model.  For stratification based on age 50 yr was used as a cut-off mark. Het.-heterogeneity.

Next, we tested if the *IFNL* polymorphisms associated with presence of asthma in different cohorts, again by stratification analysis based on both age and gender and by meta-analysis using random-effects model (Fig. 2). No significant heterogeneity in the effect between different cohorts in the sub-groups was noted. None of the sub-groups from any of the cohorts including the Polish cohort, showed any significant association with asthma. Interestingly, the older men’s sub-group showed a significant effect of the polymorphism(s) on presence/absence of asthma after meta-analysis. The minor allele (which gives rise to/linked to the allele that can express a functional IFN-λ4) had a protective effect on asthma in the older men. However, given the large sample size involved in the GERA cohort such low significance of association suggests a very small effect on the phenotype and/or phenotypic heterogeneity. Similar results were seen with a fixed-effects model (Suppl. Figure 2).

Since the GERA cohort also included data on allergic rhinitis (AR) we tested if rs12979860 and another related SNP rs8099917 associated with it (Table 6) in gender-stratified sub-groups. No significant association of either SNP with AR was evident in males or females.

**Table 6:** Association of *IFNL* locus SNPs with allergic rhinitis in the GERA cohort.

GERA cohort	Allergic Rhinitis				
SNP	Model tested	Group/sub-group	N, total; n, cases; n, controls	OR (95% CI)	p-value
rs12979860 (C/T)	Dominant (CT + TT vs CC)	All	56637; 13936; 42701	1.01 (0.97, 1.05)	0.62
		Men	22716; 4859; 17857	1 (0.94, 1.07)	0.96
		Women	33921; 9077; 24844	1.02 (0.97, 1.07)	0.51
rs8099917 (C/T)	Dominant (TG + GG vs TT)	All	Same as above	1 (0.96, 1.04)	0.91
		Men		1 (0.94, 1.07)	0.95
		Women		1 (0.95, 1.05)	0.93

GERA cohort consisted of predominantly older participants (average age 63 yr, range 18 to over 100 yr).

**Figure 3:**
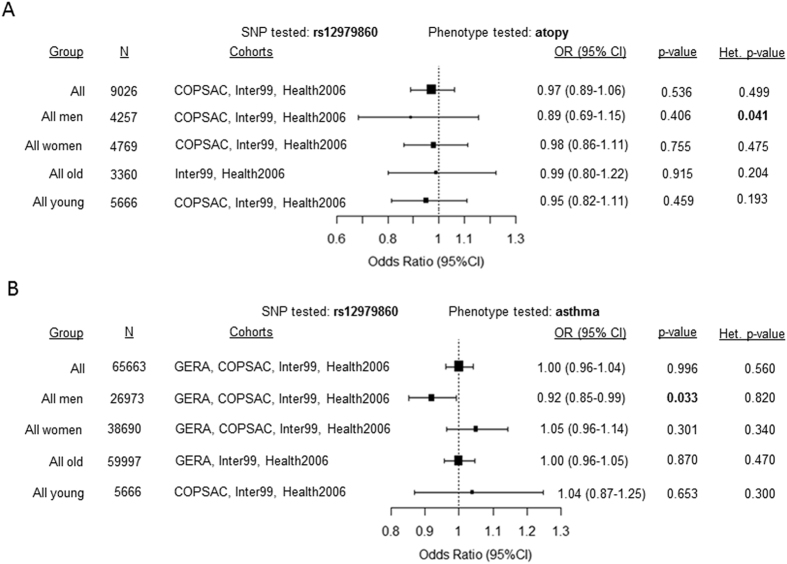
Forest plots showing the summary estimates obtained from all the participants or from different groups based on age and gender after meta-analysis of data from different cohorts for (**A**) atopy and (**B**) asthma. The association was tested with atopy or asthma and rs12979860 using a dominant model for the minor allele (CT + TT vs CC) in all cohorts except for atopy in the COPSAC cohort where an additive model (for minor allele T) was used. The cohorts included in each of the meta-analysis and the effect heterogeneity between different cohorts is shown. p-value < 0.05 was considered as significant and is in bold. Meta-analysis was performed using a random-effects model, while the results were similar using the fixed-effects model too.  For stratification based on age 50 yr was used as a cut-off mark. Het.-heterogeneity.

We extended our meta-analysis to see if the *IFNL* polymorphisms associated with atopy and asthma in: 1) all individuals irrespective of age and gender and 2) gender-specific and age-specific strata from the other European cohorts. All the cohorts included for this meta-analysis had the same SNP rs12979860 either directly genotyped or imputed and the MAFs in each of the cohorts were similar (Table 5). All studies were homogenous for the effect of the polymorphism except for the all men’s group when testing for atopy (Fig. 3A). A significant association with asthma was detected only in the sub-group of ‘all men’ (Fig. 3B) similar to the effect seen for asthma in the older men’s sub-group in Fig. 2. Since, we did not have enough participants in the younger men’s group compared to the large sample size of older men from the GERA cohort, the modifying effect of age, if any, on the association of the polymorphism with asthma could not be reliably tested.

In conclusion, the results from the Polish cohort on the association of the polymorphism(s) with atopy could not be replicated in other European cohorts while a significant association of the polymorphism(s) was seen with asthma in men/older men when data was meta-analyzed. In instances where a significant association was seen, the minor allele showed a protective phenotype.

### Functional IFN-λ4-generating ΔG allele associates with less severe features of asthma in the older women’s sub-group of the Polish cohort

We refocused our interest on the Polish older women’s sub-group where we saw a significant association of the IFN-λ4-generating polymorphism with atopy. We observed that this sub-group which comprised of both asthmatic and control older women had a majority (75%) of asthmatics (Table 4). Therefore we were interested to examine if any of the clinical features of asthma are also associated with the polymorphism (Table 7 and Table 8) in the older asthmatic women. We saw a significant association between the polymorphism and positive bronchial reversibility (or bronchodilator response, BDR) test and usage of corticosteroids (CS) for treatment (Table 8). Older women with at least one copy of the IFN-λ4-generating ΔG allele were less likely to be treated with iCS (inhaled CS) (OR = 0.35) and oCS (oral CS) (OR = 0.16) but were more likely to show a positive BDR (OR = 2.58) by univariate analysis. To test for confounders we carried out multivariate regression analysis and found that atopy could be partly mediating the association between the polymorphism and BDR (Table 8).

**Table 7:** Clinical features of asthma in older women according to rs368234815 genotypes in the Polish cohort.

Variable	Genotype		p-value
	TT/TT	TT/ΔG + ΔG/ΔG	
Age (years)	66 + /− 8,2	68,3 ± 9,9	0.156
age at asthma diagnosis (years)	47,3 ± 15,2	50,5 ± 18	0.253
asthma duration (years)	18,8 ± 14,4	18,6 ± 17,6	0.954
ACT sum (points)	17 ± 5,2	17,5 ± 5,7	0.574
MRC (points)	2,7 ± 0,9	2,6 ± 1	0.471
FeNO (ppb)	32,4 ± 27	24,7 ± 15,1	0.098
BMI	28,6 ± 5,3	27,5 ± 4,1	0.199
FEV1% pred.	76,4 ± 25	70,7 ± 25,4	0.189
FVC% pred.	89,8 ± 21,2	85,9 ± 25,4	0.345
FEV1%/FVC%	68,5 ± 14,5	67 ± 12,5	0.507

Values are presented as arithmetic means ± SD.

**Table 8:** Association of the functional IFN-λ4-generating polymorphism rs368234815 with clinical features of asthma in women > 50 yr age sub-group in the Polish cohort.

	BDR	iCS	oCS
TT/ΔG + ΔG/ΔG (n/N, %)	41/64, 64.1	61/83, 73.5	2/83, 2.4
TT/TT (n/N, %)	20/49, 40.8	54/61, 88.5	8/61, 13.1
OR crude	**2.583**	**0.359**	**0.163**
95% CI	**1.203–5.55**	**0.142–0.907**	**0.033–0.8**
p	**0.01**	**0.03**	**0.02**
OR adjusted*	**4.651**	**0.112**	0.146
95% CI	**1.414–15.15**	**0.02–0.619**	0.013–1.594
p	**0.01**	**0.01**	0.10
OR adjusted**	2.262	**0.248**	0.227
95% CI	0.975–5.235	**0.081–0.751**	0.041–1.237
p	0.05	**0.01**	0.08

Dominant model of inheritance for the minor allele (TT/ΔG + ΔG/ΔG vs TT/TT) was tested; No significant results were seen under the recessive model. *OR adjusted for age, age at asthma diagnosis, FeNO, BMI, FEV1% and atopy; **OR adjusted for atopy. p < 0.05 was considered significant and is shown in bold.

In summary, results from the Polish cohort demonstrated that IFN-λ4-generating ΔG allele protected a subpopulation of asthmatic patients, specifically older women, from allergic sensitization. The IFN-λ4-generating ΔG allele also associates with less inhaled and oral CS usage, suggesting that it may be related with lesser disease severity in this group of severe asthmatics (78% of overall cases had either partially controlled or uncontrolled asthma, Table 1). Further, it goes on to suggest that the ΔG allele carriers possibly had reduced airway inflammation compared to the pseudogenizing TT/TT genotype carriers.

### Inherent differences between the discovery and replication cohorts may have been responsible for non-replication of our results from the Polish cohort

The association of *IFNL* polymorphism with atopy in the Polish older women’s cohort survived correction for multiple testing that was carried out as a measure to negate the limitation of post-hoc analysis of data (Table 3). Further, the results from the Polish study are not due to a ‘cohort effect’ since the association of the polymorphism with atopy is within the older women’s ‘cohort’ and not between an older and a younger ‘cohort’. The association of the polymorphism in older women was not just with atopy but also translated to association with some important determinants of severity of asthma like BDR and CS usage. Even though we did not carry out multiple testing correction for our association tests with the polymorphism and clinical features of asthma (Table 8) we feel the phenotypes are not independent of the atopic sensitization phenotype that we identified during our initial analysis (Tables 3 and 4), and therefore less likely to be false positives. For example, atopy-related eosinophilic inflammation and increased CS usage frequency among patients may be correlated^24^; this is apart from the fact that atopy was a confounder with the association involving bronchial reversibility tests (Table 8). Therefore the results from the Polish study regarding association of the polymorphism with atopy and clinical features of asthma in older women, seem to be consistent with each other.

It is important to analyze and interpret our results from the Polish study in the context that they were not replicated in other cohorts of the European population. It should be noted that the replication study had several limitations. Firstly, the findings we made in the Polish cohort were from a hospital-based case-control design while all the three replication cohorts (except COPSAC cohort) were population-based (Table 5). Secondly, 72% of the Polish cohort (among the 384 subjects with information on atopy) were asthma cases and specifically the older women’s sub-group in this cohort had substantially more (75%) asthmatics than controls while the replication cohorts had 16% asthmatics among older men and women in GERA cohort (10% in AR-negative controls and 34% in AR-positives; AR-negative controls were 77% and AR -positive were 33% in the cohort); 9.6% and 11.6% of older women had asthma in the Inter99 and Heath2006 cohorts respectively. It is possible that the association with atopy in the Polish cohort had an underlying link with asthma that could not be accounted for in the replication study. In support of this, significance of association decreased when data was adjusted for asthma status in the older women’s sub-group in the Polish study (Table 4). Thirdly, the Polish cohort was enriched with severe asthmatics (22% controlled, 30% partly controlled and 48% uncontrolled according to GINA 2016; up to 65% of patients were in medium or high dose iCS; 60% were on long acting β-agonists; Table 1) while this information was not available for the replication cohorts. Fourthly, similar methods were not used to test for the phenotypes in all the cohorts (Ex. SPT in the Polish cohort and serum specific IgE in the Danish cohorts to test atopy). While in the discovery cohort that had a hospital-based case-control design, we saw a significant association of the polymorphism with atopy (in an asthma-enriched background) and severe asthma features in older women, in the replication study we tested for association with atopy, allergic rhinitis and presence/absence of asthma in a population-based cohort. These discrepancies in endpoint phenotypes and the intrinsic differences in the composition of the Polish and the replication cohorts may have led to non-replication of the findings.

Our findings from the Polish cohort need to be further validated in well-designed replication studies and functional studies. It is likely that the functional IFN-λ4-generating dinucleotide variant rs368234815 may associate with a specific endotype of severe asthma in older patients. It is possible that a sustained course of inflammation of the airways that happens in older asthmatics, likely in response to viral infections over a period of time, will lead to an environment conducive for the penetrance of the genetic effect of *IFNL* variants in asthma. Since the Polish cohort had >60% women asthma patients and was enriched with older women (Table 1) we saw strong association in the older women’s sub-group (Fig. 1 and Table 3). We hypothesize that presence of IFN-λ4-generating ΔG allele may be beneficial for an elderly female asthma patient by protecting the airways from increased inflammation associated with virus-induced asthma exacerbations. Although, no direct association of the allele with history of asthma exacerbations or hospitalizations was revealed (data not shown), it should be noted that the Polish asthma cohort represented a group of difficult-to-control asthmatics (only 22% had well-controlled disease according to GINA criteria; Table 1). Along these lines, association of the allele with the presence of acute airway reversibility in response to inhaled beta2- agonists may indirectly reflect less severity of the disease and/or lower airway remodeling in those patients that carry an IFN-λ4-generating ΔG allele. It remains to be established if the effect of the functional IFN-λ4-generating variant on asthma control and severity is also valid in older men. We did see a protective effect of the minor allele rs12979860 (which tags the IFN-λ4-generating allele) against asthma in older men/all men after meta-analysis of data (Figs 2 and 3). However, the significance of association is low given the large sample size tested.

Further, it remains to be verified if rs368234815 is the functional variant behind this association in atopy/asthma or other *IFNL* SNPs may also contribute to the phenotype by altering the levels of IFN-λ3 expression similar to recent observations in hepatic inflammation and fibrosis^25^. Future studies aimed at understanding the functional role of IFN-λ4 in regulating inflammation of the airways are required to understand the mechanism behind the association that we identified in the Polish study. Alternately, this finding could be a false positive result, which also has to be confirmed by doing a well-designed replication study first in another Polish cohort and later in other populations. Nevertheless, our comprehensive analysis with sufficiently large sample sizes has established that an important candidate gene locus consisting of the immunomodulatory type III IFNs does not associate with atopy and AR in the general population (Fig. 3A and Table 6). The association could be more subtle and with specific endotypes related to inflammation of the airways in asthma. The protection to asthma seen in men/older men (Figs 2 and 3) is nominal and further studies can aim at validation of this result in more specific endotypes based on severity of asthma. A fully functional IFN-λ4 may be associated with protecting the airways from inflammation in certain endotype(s) of the disease, specifically in older asthmatics. While further studies are needed to understand this association, whether these endotypes are a result of virus-induced stimuli, also remains to be examined. A previous report documented a strong positive association of allergy^26^, interestingly more pronounced in females than males, with the minor allele of rs12979860 (which is in strong LD with rs368234815^6^) in a pediatric cohort. Our results from the Polish cohort, in contrast, show that the IFN-λ4-generating ΔG minor allele may protect older women from atopy rather than contributing to it. The reasons for this paradox remain to be investigated but may likely involve complex epistatic effects mediated by other innate or adaptive immunity genes and age-dependent changes in Th1/Th2 balance during the transition from infancy to adulthood. Interestingly, age and gender are known to interact and influence the association of rs12979860 with another inflammatory condition of Th2-origin, fibrosis, in chronic HCV infections^27^.

## Material and Methods

### Polish study

Both the controls (N = 111) and asthmatics (N = 326) belonged to same ethnicity (local Polish population) and geography (residents of central Poland). Asthma control was assessed according to GINA 2016 (global initiative for asthma) guidelines and atopy was defined as presence of a positive skin response (weal diameter >3 mm) to at least one of a panel of common inhalant allergens applied as a skin-prick test (SPT). Evaluation also included a questionnaire, FeNO (fractional exhaled nitric oxide) measurement, spirometry and reversibility test with 400 µg salbutamol MDI performed in 280 patients. Genomic DNA was isolated from EDTA-treated whole blood samples using the Qiagen blood genomic DNA mini kit. A competitive allele-specific polymerase chain reaction (PCR) (KASP, LGC Genomics, UK) was used to genotype the functional IFN-λ4-generating SNP rs368234815; the assay was carried out in a StepOnePlus real-time PCR machine (Applied Biosystems, UK). The study was approved by the local Bioethics Committee (document no. RNN/121/12/KE) and all study subjects provided an informed written consent. All methods were carried out in accordance with relevant guidelines and regulations stipulated by the Medical University of Lodz and/or other relevant authorities under it. Further, the university granted approval for all the experimental protocols performed in this study.

Since the dominant and recessive models of inheritance have been reported previously for the *IFNL* SNP association with various diseases^5^, we used both these models to test for association with various phenotypes in our study. Statistical analyses of data included log-linear (for testing interactions between different variables) and logistic regression (for multivariate analysis); goodness-of-fit was tested using Pearson’s chi-square test or two-tailed Fischer’s exact test. All statistical analyses were performed using Statistica 12.5 PL; p-value of < 0.05 was considered statistically significant unless specified.

### Inter99 and Health2006 study

The Health2006 Study took place from 2006 to 2008 and included a random sample of 7,931 Danish (Danish nationality and born in Denmark) men and women aged 18–69 years invited to participate in a health examination. The Inter99 Study is a randomised controlled trial (CT00289237, ClinicalTrials.gov) aiming to investigate the effects of a lifestyle intervention on cardiovascular disease (N = 61,301). The details of these two study cohorts on genotyping and data collection on atopy and asthma are described elsewhere^19, 20^. The Health2006 Study and the Inter99 Study were approved by the Ethics Committee of Copenhagen County and the Danish Data Protection Agency. All participants gave their informed consent, and all methods were carried out in accordance with relevant guidelines and regulations. The Health2006 and Inter99 datasets generated during and/or analysed during the current study are not publicly available due to ethical and legal reasons since public availability may compromise participant privacy, and this would not comply with Danish legislation. Requests for data should be addressed to Professor Allan Linneberg. Access to data will be provided in accordance with the Danish Data Protection Agency.

### GERA study

GERA cohort data was obtained through dbGaP under accession phs000674.v1.p1. The Resource for Genetic Epidemiology Research on Aging (GERA) Cohort was created by a RC2 “Grand Opportunity” grant that was awarded to the Kaiser Permanente Research Program on Genes, Environment, and Health (RPGEH) and the UCSF Institute for Human Genetics (AG036607). The RC2 project enabled genome-wide SNP genotyping (GWAS) to be conducted on a cohort of over 100,000 adults who are members of the Kaiser Permanente Medical Care Plan, Northern California Region (KPNC), and participating in its RPGEH. The resulting GERA cohort is composed of 42% of males, 58% of females, and ranges in age from 18 to over 100 years old with an average age of 63 years at the time of the RPGEH survey (2007). A subset of 62,281 subjects from European ancestry was quality controlled (QCed) and analyzed. A 3-step QC protocol was applied using PLINK and included 2 stages of SNP removal and an intermediate stage of sample exclusion. The exclusion criteria for genetic markers consisted on: proportion of missingness ≥0.05, HWE p-value ≤ 1 × 10–20 for all the cohort, and MAF <0.001. This protocol for genetic markers was performed twice, before and after sample exclusion. For the individuals, we considered the following exclusion criteria: gender discordance, subject relatedness (pairs with PI-HAT ≥ 0.125 from which we removed the individual with the highest proportion of missingness), variant call rates ≥0.02 and population structure showing more than 4 standard deviations within the distribution of the study population according to the first seven principal components. After the QC analysis, 56,637 subjects remained for genotype imputation and association testing.

We performed a two-stage imputation procedure, which consisted in pre-phasing the genotypes into whole chromosome haplotypes followed by imputation itself. The pre-phasing was performed using the SHAPEIT2^28^ tool, IMPUTE2^29^ for genotype imputation and the SNPTEST (https://mathgen.stats.ox.ac.uk/genetics_software/snptest/snptest.html#introduction) for association testing. In this work we used 1000 G Phase 3 haplotypes (October, 2014) as a reference panel to infer ungenotyped variants. After genotype imputation, variants with info score < 0.7 and MAF < 0.001 were removed. Association testing with SNPTEST tool was performed using an additive and dominant logistic regression model adjusting by the 7 derived principal components, age distributed in 14 groups and sex. Moreover, variants with HWE p.value ≤ 1 × 10^−6^ for controls were removed. The diagnostic criteria for allergic rhinitis were based on the following ICD9 codes: 477, 477.0, 477.1, 477.2, 477.8, 477.9 (https://www.ncbi.nlm.nih.gov/projects/gap/cgi-bin/GetPdf.cgi?id=phd004308).

### COPSAC study

The Copenhagen Prospective Studies on Asthma in Childhood (COPSAC2000) is a clinical study comprising 411 children with high risk of asthma born to asthmatic mothers. Information on Doctor diagnosed asthma was available for parents of COPSAC2000 study which was used in the current analyses. The COPSAC study is described in detail elsewhere^21^. Genotyping of parents was performed on the Illumina Infinium II HumanHap550 BeadChip and has been described previously^22^. All participants gave their informed consent. The Ethics Committee for Copenhagen and the Danish Data Protection Agency approved this study.

### Meta-analysis

R programing using the function ‘rma’ from ‘metafor’ R package (R Core Team, 2015) was used. Pooled data were analysed by using a random-effects model (using DerSimonian-Laird’s method). The random-effects model was chosen even when effects were homogenous across cohorts since one cohort (GERA) had overwhelmingly large sample size compared to other cohorts and also the cohorts were not uniform in their characteristics (Ex. the Polish cohort was a hospital-based case-control study while the other cohorts were population-based). The significance of the pooled OR was determined by the z-test. Heterogeneity between studies was assessed using the Chi-squared based Cochran’s Q-test.

## Electronic supplementary material

Supplementary Information
