# Supplementary material for: A functional IFN-λ4-generating DNA polymorphism could protect older asthmatic women from aeroallergen sensitization and associate with clinical features of asthma
Source: Sci Rep. 2017 Sep 5;7:10500. doi: 10.1038/s41598-017-10467-y (PMC5585370; doi:10.1038/s41598-017-10467-y)

A functional IFN-λ4-generating DNA polymorphism could protect older asthmatic women from aeroallergen sensitization and associate with clinical features of asthma

Sreedhar Chinnaswamy[1, 7*], Aleksandra Wardzynska[2], Malgorzata Pawelczyk[2], Joanna Makowska[2, 3], Tea Skaaby[4], Josep M. Mercader[5], Tarunveer S. Ahluwalia[6], Niels Grarup[11], Marta Guindo-Martinez[5], Hans Bisgaard[6], David Torrents[5, 10], Allan Linneberg[4,8,9], Klaus Bønnelykke[6], Marek L. Kowalski[2, 7]

Suppl. Fig. 1: Fixed-effect meta-analysis

A) Association of *IFNL* polymorphisms with atopy in older men

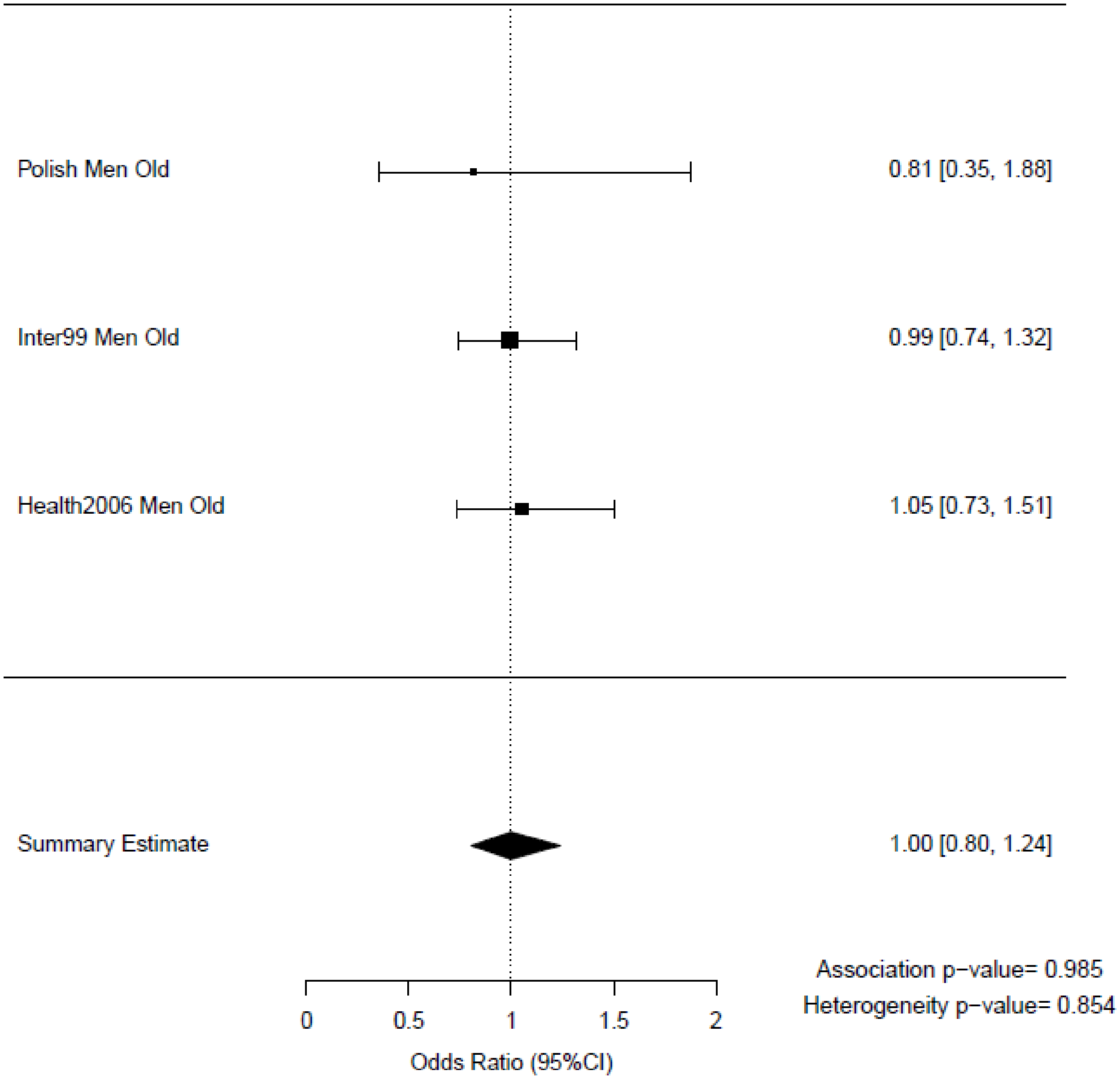

Suppl. Fig. 1: Fixed-effect meta-analysis
B) Association of *IFNL* polymorphisms with atopy in younger men

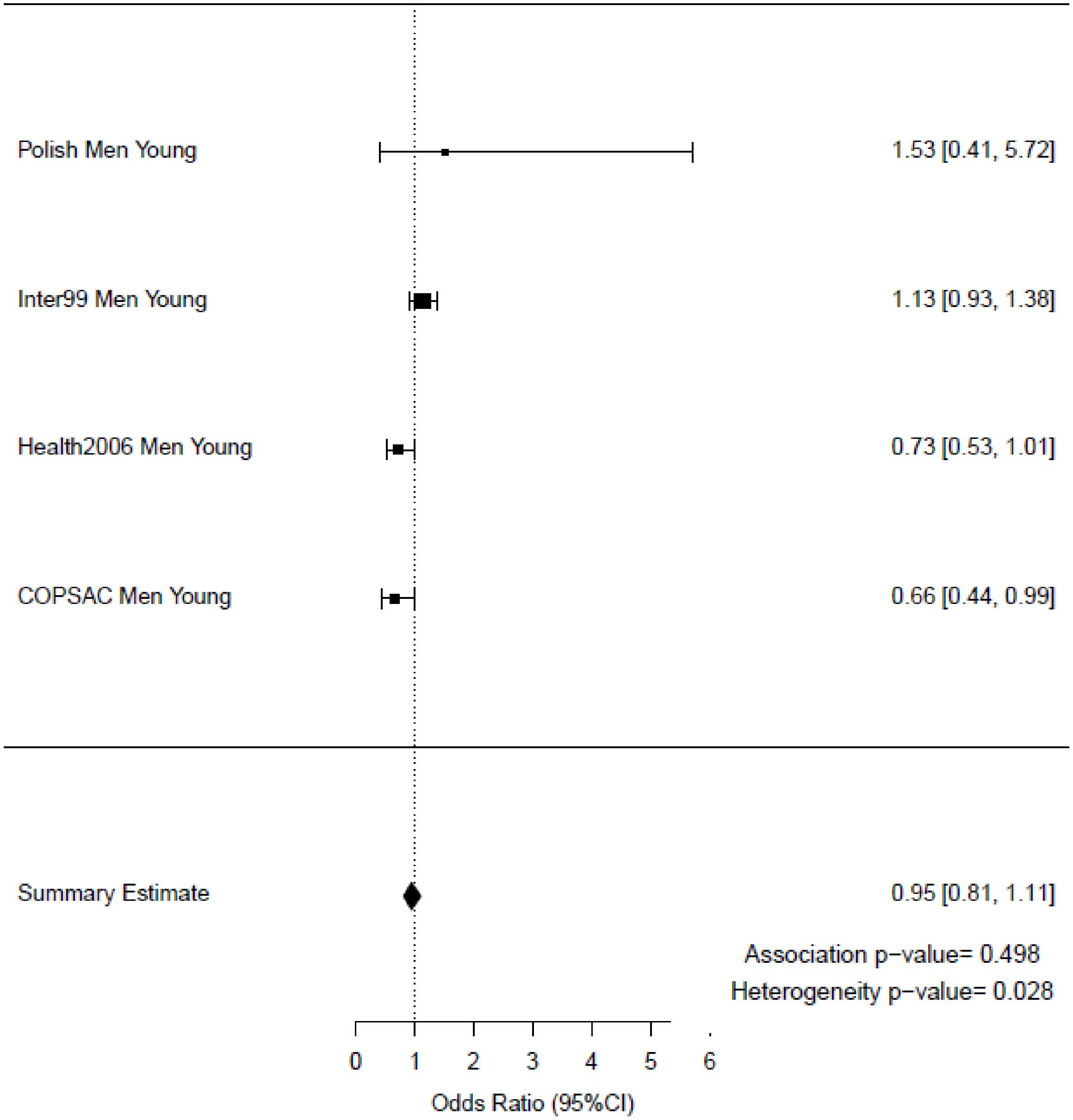

Suppl. Fig. 1: Fixed-effect meta-analysis
C) Association of *IFNL* polymorphisms with atopy in older women

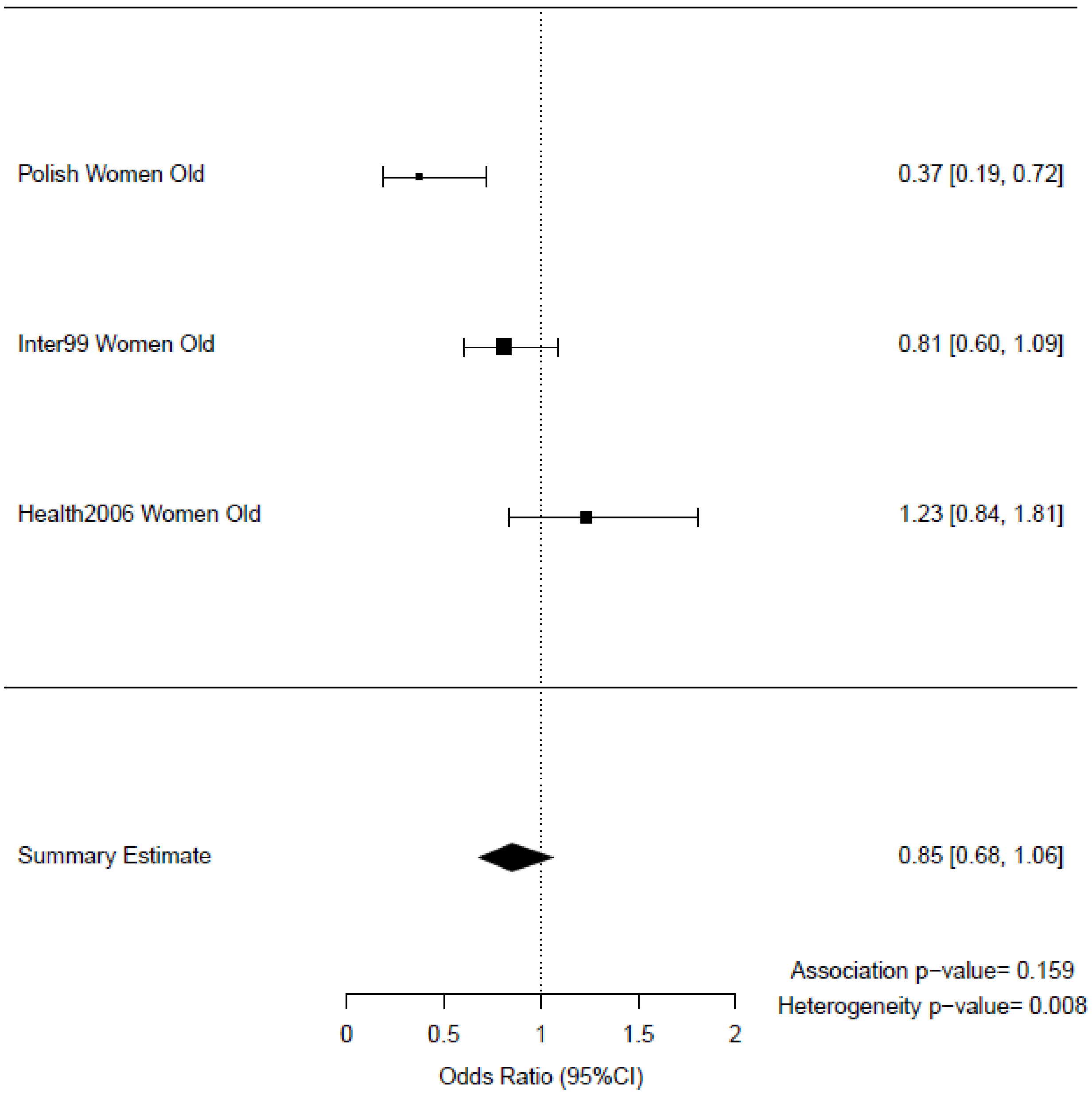

Suppl. Fig. 1: Fixed-effect meta-analysis
D) Association of *IFNL* polymorphisms with atopy in younger women

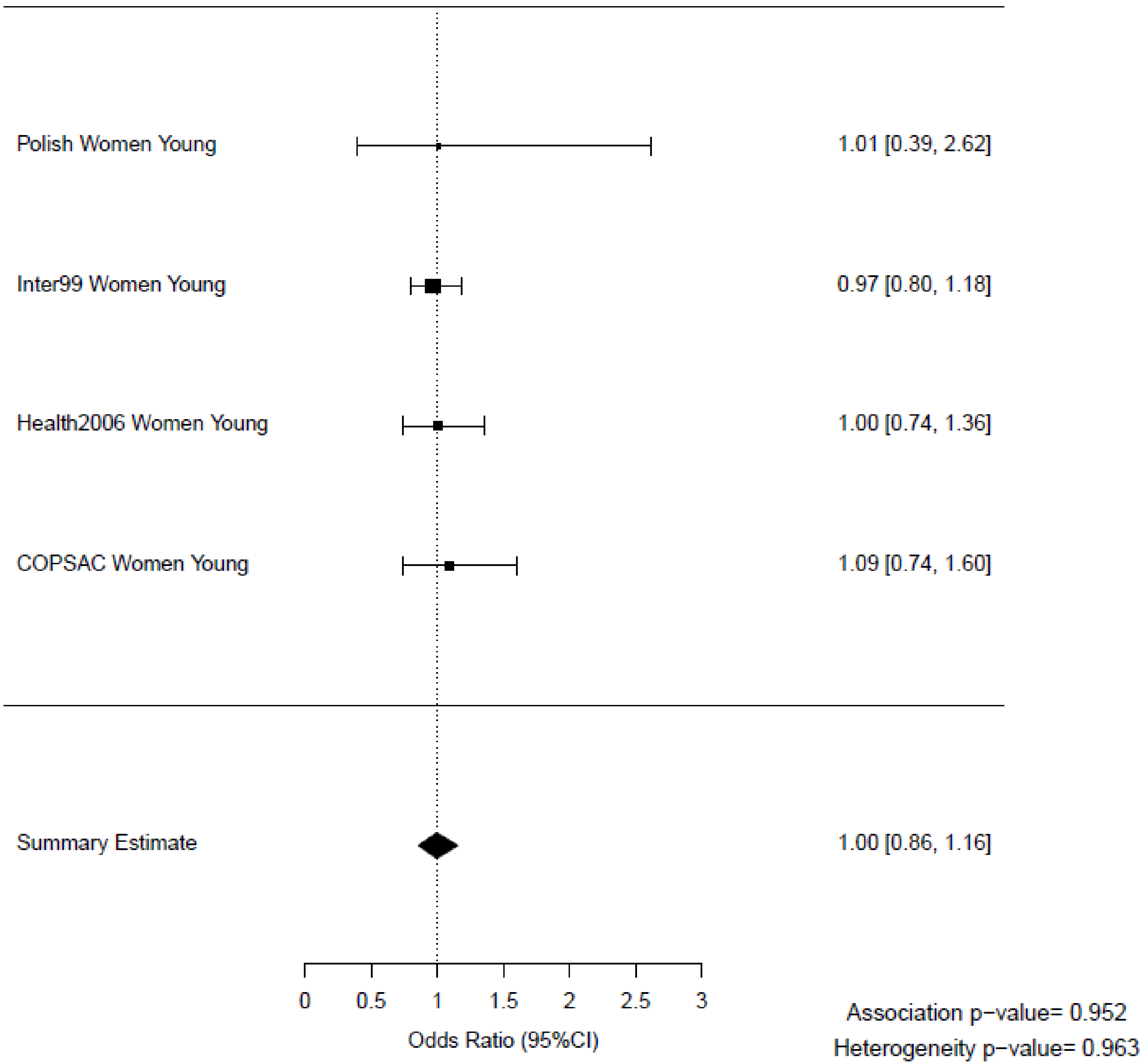

Suppl. Fig. 2: Fixed-effect meta-analysis
A) Association of *IFNL* polymorphisms with asthma in older men

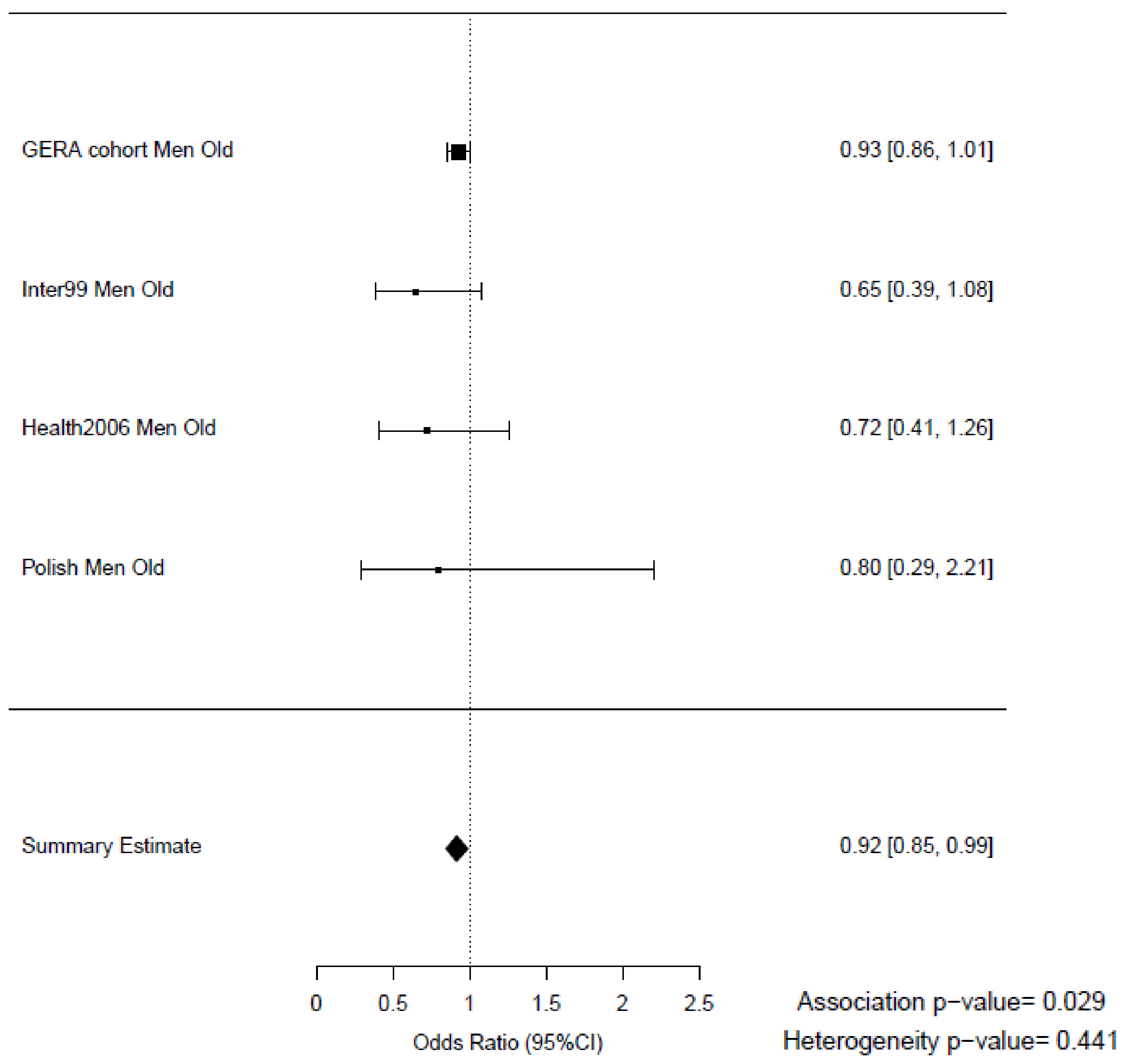

Suppl. Fig. 2: Fixed-effect meta-analysis

B) Association of *IFNL* polymorphisms with asthma in older women

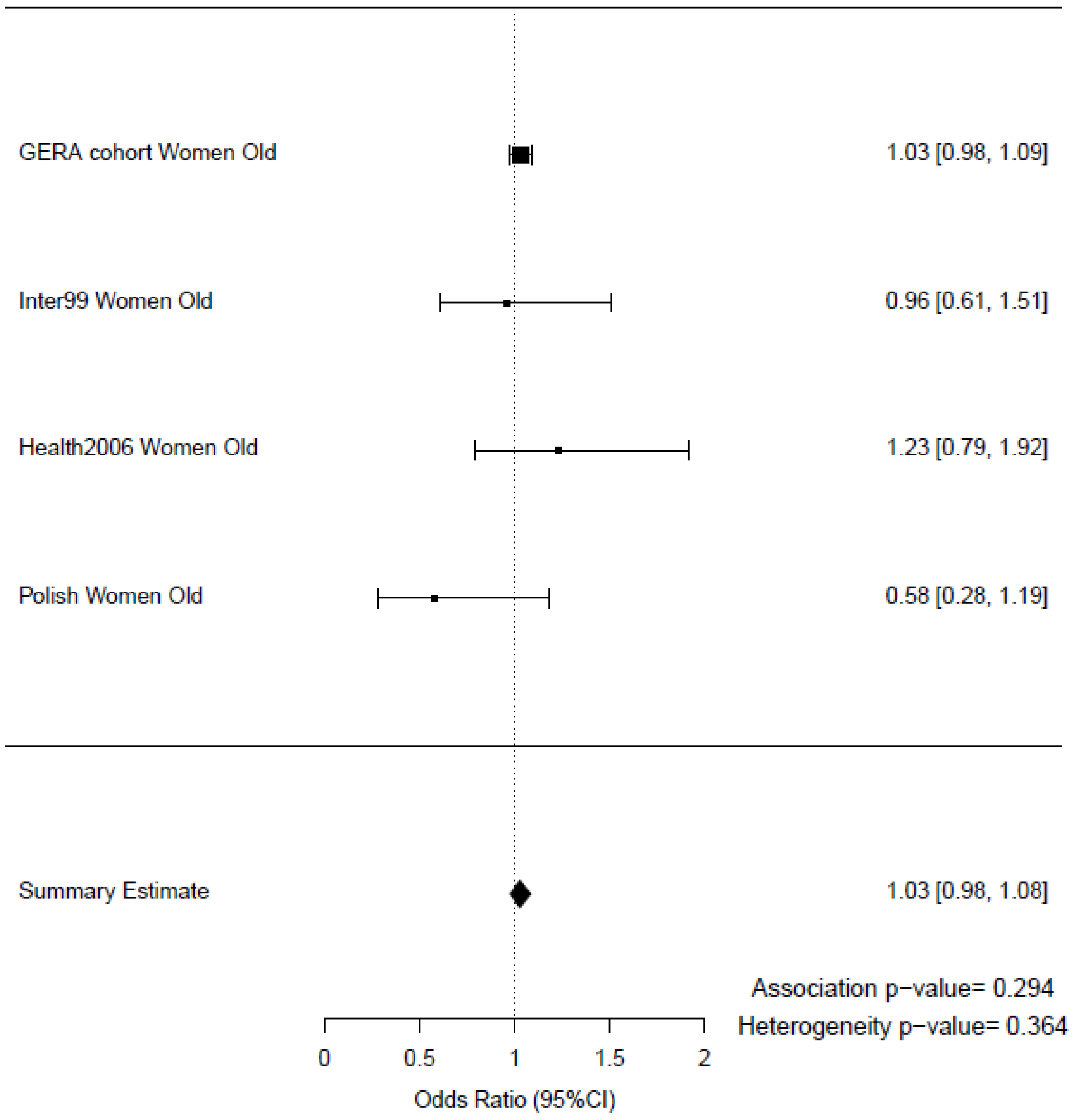

Suppl. Fig. 2: Fixed-effect meta-analysis
C) Association of *IFNL* polymorphisms with asthma in younger men

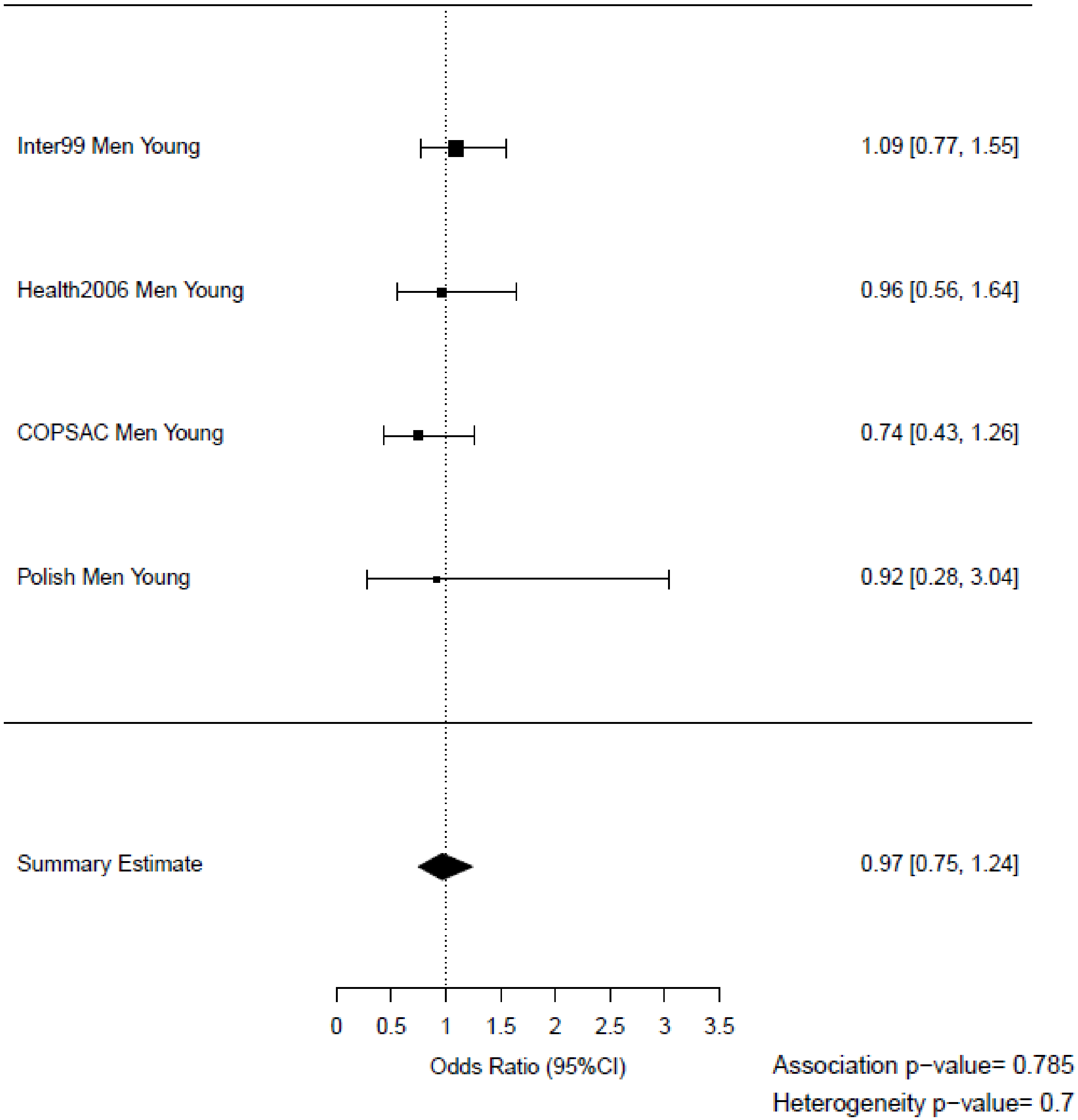

Suppl. Fig. 2: Fixed-effect meta-analysis
D) Association of *IFNL* polymorphisms with asthma in younger women

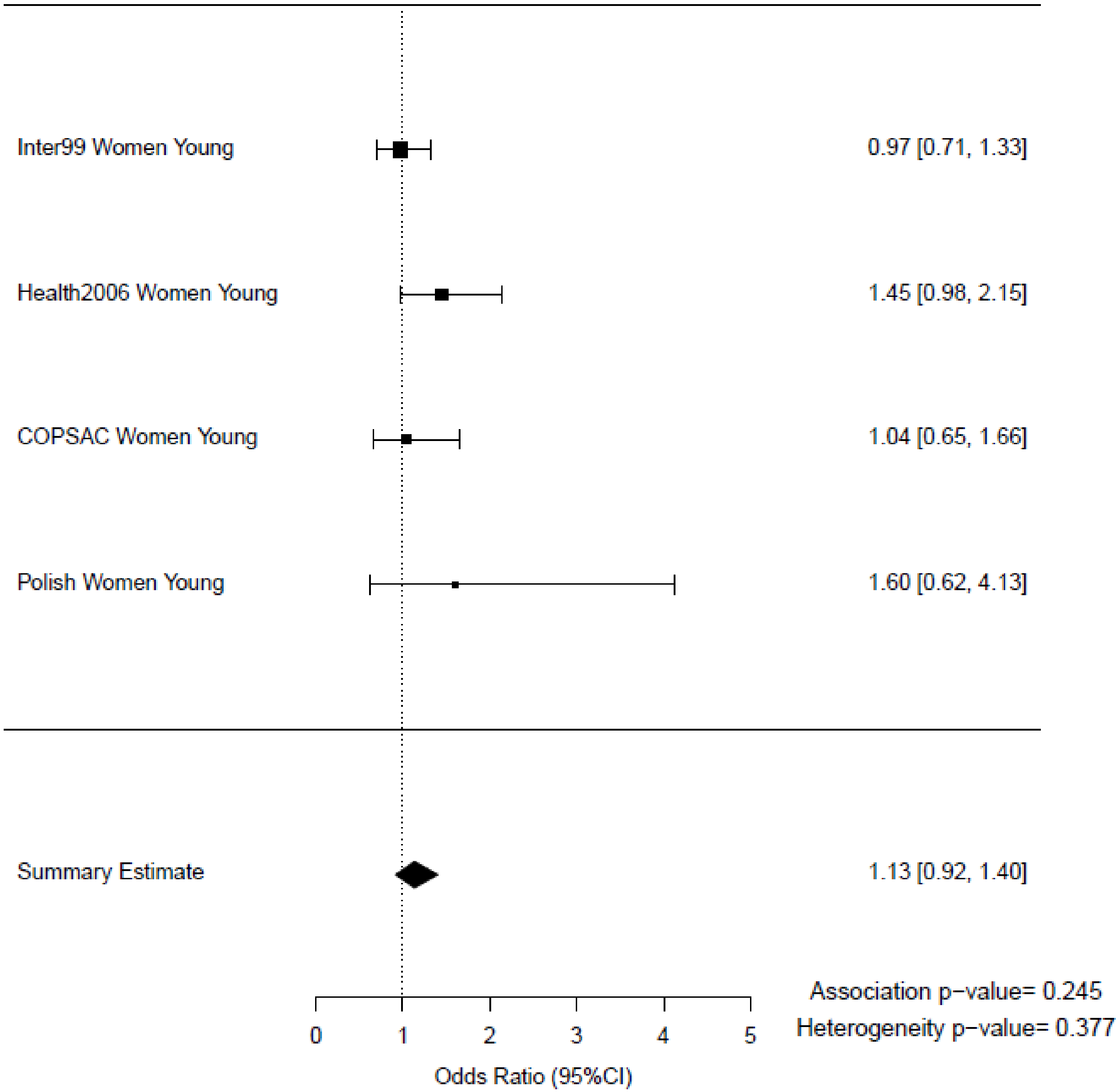

Supplement: Supplementary file 1 — Supplementary Information [file 41598_2017_10467_MOESM1_ESM.pdf]
